# Factors Influencing the Creativity of Chinese Upper-Secondary-School Students Participating in Programming Education

**DOI:** 10.3389/fpsyg.2021.732605

**Published:** 2021-12-23

**Authors:** Jun Liu, Xue Sun, Meng Sun, Yan Zhou, Xinyue Li, Jinbo Cao, Zile Liu, Fei Xu

**Affiliations:** ^1^College of Education, Capital Normal University, Beijing, China; ^2^Xinyu School Affiliated to Beijing Normal University, Xinyu, China; ^3^College of Teacher Education, Capital Normal University, Beijing, China

**Keywords:** mainland china, creativity, programming education, influencing factors, programming teaching, programming learning

## Abstract

**Purpose:** This study explored whether instructional characteristics, learner characteristics, family socioeconomic status, and gender influence creativity in the context of programming education in China.

**Methods:** A total of 851 upper-secondary-school students in Beijing, China, were surveyed using the Creativity Scale, Programming Learning Scale, Programming Teaching Scale and Family Socioeconomic Status Questionnair*e*. SPSS (version 22) was used for correlation analysis, *t*-test and regression analysis.

**Results:** (1) Teachers’ programming teaching method and management; students’ programming learning approach, attitude, and engagement; gender; and family economic capital were all significantly associated with creativity. (2) There were significant differences between males and females in terms of creativity, programming learning approach and programming learning attitude. (3) Learner attitudes, engagement, and approach, and their family economic capital, were strong predictors of creativity, with the strongest influence of learners’ attitudes to programming learning and weaker influence of family economic capital.

**Conclusion:** The main factors that influence creativity in the context of programming education are programming teaching method, programming teaching management, programming learning approach, programming learning attitude, programming learning engagement and family economic capital. Among these, learner factors (attitude, engagement, and approach) and family economic capital are the key factors influencing creativity. These findings provide a basis for improving the creativity of Chinese programming learners and inspire teachers to consider learner factors and gender differences as they design and manage their instruction. Furthermore, the influence of family economic capital on the creativity of learners cannot be ignored.

## Introduction

As an important aspect of learning, creativity is an indicator of student development. McWilliam (2009) identified creativity as a key learning outcome of our time and the core business of education. Also, in 2018, the OECD (2018) in its publication “*Learning Framework for 2030*” mentioned that creativity is a necessary skill for learners, and recommend it as an educational focus. Similarly, Zhang et al. (2021) argued that creativity is an important component of any educational programming. As Shu et al. (2020) stated, creativity is essential for every culture and society, which is required to solve new problems for students in the twenty-first century (de Vries, 2021; Kozhevnikov et al., 2021).

Increasing evidence confirms the importance of programming education in creativity development. The American New Media Alliance pointed out that programming will gradually become a key element promoting basic education (Sun and Li, 2019), which enables learners to create new strategies to solve problems and to test innovative solutions in all disciplines (Saritepeci, 2019). In other words, programming education takes place in a technology-enhanced environment (Hung and Sitthiworachart, 2019), which provides the most direct pathway for the development of thinking skills (Fu et al., 2021). Furthermore, experts agreed that fun programming methods can develop creativity (Tengler et al., 2020). Similarly, Noh and Lee (2020) demonstrated that programming itself is a creative activity through an 11-week programming course experiment, which can stimulate students more creative.

Although few scholars have explored what factors are associated with the development of creativity in the context of programming education, studies have shown that it is related to certain characteristics of learners. For example, Campos Cancino and Moreno Minguez (2020) argued that family factors are the basis for learners’ cognitive and emotional development, which influence the development of learners’ creativity. Similarly, Yang et al. (2020) used data analysis to find that the socioeconomic status of college students’ families predicted creativity. Liang et al. (2021) also found that respondents’ self-rated creativity is positively related to family socioeconomic status in an investigation among Chinese adolescents aged 9–14. In addition, Zhang et al. (2020) argued that learners’ gender also affects creativity; specifically, boys outperform girls in terms of originality of creativity, but girls outperform boys in terms of abstraction. Also, Noh and Lee (2020) study demonstrated gender differences in learner creativity.

Besides, promoting learners’ creativity by programming education has certain relation with the effectiveness of programming instruction. Research has shown that there are at least two major factors affecting the effectiveness of programming instruction: teacher teaching and student learning. In the case of programming instruction, teachers’ teaching methods and management can affect the effectiveness of programming instruction (Kiss and Arki, 2016; Bi and Shi, 2019; Wu and Hao, 2019). For example, Tang et al. (2017) demonstrated through experiments that using flipped class approach in programming courses can improve students’ cognitive and competence levels. Again, Du and Liang (2011) demonstrated through validated factor analysis that the role of teachers in managing the classroom was a factor in the effectiveness of teaching and learning. In terms of student programming learning, existing research has found that learners’ attitudes toward learning programming (Durak, 2018b), learning approach (Tan and Lee, 2017), and learning engagement (Tian and Wu, 2018) also influence learning outcomes.

Therefore, a more comprehensive perspective is needed to examination the influencing factors of students’ creativity development, which contains three aspects: programming learner’s family factors, teacher’s programming teaching factors and programming learners’ individual factors. Specifically, we propose the following research hypotheses.

H1: The family economic capital of Chinese teenagers participating in programming education affects their creativity.

H2: The family cultural capital of Chinese teenagers participating in programming education affects their creativity.

H3: The family social capital of Chinese teenagers participating in programming education affects the creativity.

H4: The gender of Chinese teenagers participating in programming education influences their creativity.

H5: Teacher teaching of Chinese teenagers participating in programming education affects their creativity.

H6: Characteristics of Chinese teenagers participating in programming education affect their creativity.

## Literature Review

### Creativity

Anything new arises from *creativity* (Noh and Lee, 2020). Creativity is the ability to create new products or ideas that are valuable and useful (Woodman et al., 1993; Shalley et al., 2016; Qiang et al., 2020; Hou et al., 2021; Zhang et al., 2021). Generally, this ability is reflected in behaviors such as inventing, designing, inventing, creating, and planning (Guilford, 1950) and is characterized by fluency, flexibility, novelty, and refinement (Kupers et al., 2018; Kim et al., 2019; Rao et al., 2021) and is the result of the interaction between capacity, process and environment results (Gu et al., 2021).

### Family Socioeconomic Status

*Family socioeconomic status* comprises an individual’s or family’s property income, the educational level of parents, and parental occupation (Wiederkehr et al., 2015; Yang et al., 2020). Family property income is defined as the capital, which is used to purchase material goods like household goods, books, mobile phones, and computers. Parental occupation refers to the resources acquired through parents’ social interactions and relationships, including their work or occupation (Entwisle and Astone, 1994).

Some studies have found that the family socioeconomic status affects learners’ creativity. For instance, Zhang et al. (2018) studied a sample of 955 students and found that family socioeconomic status is significantly related to creativity, though students with low family socioeconomic status face limitations in creativity while solving problems. Similarly, Lebuda and Csikszentmihalyi (2020), using a grounded-theory methodology, found that family factors affect the development of creativity. Likewise, Yang et al. (2020) showed a significant positive association between family socio-economic status and creativity in a survey of students at ten universities. Also, Jankowska and Karwowski (2019) related the level of creativity of children to family socioeconomic status.

### Programming Education—Instruction

In programming education, *instructional approach* refers to the purposeful and organized way in which teachers guide students in acquiring knowledge and skills in computer programming, including aspects such as curriculum content, learning environment, teaching strategies, curriculum, and evaluation of student achievement.

Schooling is seen as a major site for creativity development (Kong et al., 2013; Gu et al., 2021), and some experts have found a relationship between teaching and creativity. For example, Lin et al.’s (2017) study with 104 students showed that exploratory education can have a significant impact on creativity development. Likewise, Huang et al. (2021) argued that adaptive teaching models were effective in enhancing students’ creativity. In addition, a survey of 872 teachers and 944 students by Burayeva et al. (2020) showed that effective management strategies were beneficial in stimulating students’ creative potential. Similarly, problem-based teaching (Sidek et al., 2020), project-based teaching (Wu and Wu, 2020), and active teaching methods (Vokic and Aleksic, 2020) all help to stimulate students’ creative processes and foster their creativity.

### Programming Education—Learning

Learners acquire programming knowledge and skills through a process that involves their learning approach, learning attitude and their learning engagement. Researchers have found that learner engagement, as the initial condition for learning, plays an important role in learning computer programming (Durak, 2018a), and that negative attitudes toward learning how to program can affect engagement in programming courses (Durak, 2020).

For instance, Zuo et al. (2019) found that learners’ perceptions can influence their creativity. Flipped learning strategies and self-directed learning styles can enhance learners’ creative performance (Hsia et al., 2021; Shafait et al., 2021). Again, studies have found a strong correlation between individual affective attitudes and creativity (Silva and Coelho, 2019; Hernandez-Jorge et al., 2020). Furthermore, an empirical study conducted by Yang (2021) on 466 practitioners also showed a positive correlation between personal work engagement and creativity. Similarly, Sun (2020) survey of 652 university students showed that student engagement moderated the relationship between social media use and student creativity.

### Gender

Currently, although there are many studies on gender differences in creativity, scholars have not reached agreement regarding the influence of gender differences on creativity. Taylor and Barbot (2021) found that no significant difference between the scores of males and females on creative drawing tasks. Similarly, Koronis et al. (2019) findings revealed no correlation between gender and creativity. In contrast, Zhang et al. (2020) suggested that males performed better in some aspects of creativity.

## Materials and Methods

### Participants

Participants were drawn from four public schools in Beijing, China, the region that first initiated programming education. A combination of convenience and random sampling method was used for this study. First, the researchers chose one school each in Xicheng, Haidian, Chaoyang, and Shunyi Districts of Beijing, China. The four schools had been teaching computer programming for a long time and served the same grade levels. Second, a random sample of upper-secondary-school-students from each school was selected to complete the questionnaire in about 15 min for the study. A total of 851 students (aged 16–18) took part in the study and completed the questionnaire. Of the respondents, 405 (47.59%) were male and 492 (52.41%) were female.

In order to ensure the authenticity and reliability, this study used paper questionnaires in the classroom environment with the permission of programming teachers and participants. Before the survey, all participants knew the purpose of the study and participated voluntarily. Moreover, the researchers explained that they can leave the classroom at any time without any reason, and all information would be kept confidential. It is worth noting that all participants agreed to use their anonymous data.

### Measures

The instrument used in this study contains five sections: demographic information, Creativity Scale, Programming Learning Scale, Programming Teaching Scale, and Family Socioeconomic Status Questionnaire. The demographic information section collected data on gender and age. All three scales and the questionnaire were originally developed in English and were translated into Chinese for use in this study. Given that Brislin (1970) demonstrated that checking the quality of back-translations is valid, we adopted his method and back-translated the instrument: one researcher translated the measures from English to Chinese, and the second researcher translated the Chinese version back to English, and finally, the third researcher compared the three versions (original, translation, and back translation) to determine the equivalence among them.

#### Creativity Scale

The Creativity Scale is based on the Adaptor and Innovator Scale (Kirton, 1976), which is currently the most widely used measure of creativity. The scale contains 32 items, including three dimensions: originality, organization and appropriate respect for authority and rules. Sample items were “I usually have original ideas about problems “and” I often break the rules when doing things.” Each item is scored on a 5-point Likert scale: 1 means *completely disagree* and 5 means *strongly agree*. Scores range between 32 and 160, and the higher the score, the more the individual’s creativity tends toward innovation. The Cronbach’s alpha from the original study was reported as 0.88. Before collecting the formal data, we tested 160 students with Cronbach’s alpha of 0.969. In addition, exploratory and confirmatory factor analysis revealed factor loading of the scale was between 0.5 and 0.87, which shows the good reliability and validity. In this study, the alpha was 0.979.

#### Programming Learning Scale

According to the OECD (2009), we have compiled the Programming Learning Scale based on the characteristics of programming education. The scale consists of 22 items and is divided into three dimensions: programming learning approach, programming learning attitudes and programming learning engagement. Scores range from 22 to 110: the higher the score, the more effective the learner at learning computer programming. Before beginning formal data collection for this study, we tested the scale with 160 students, the Cronbach’s alpha was 0.917, and the factor loading was between 0.55 and 0.88. In this study, the alpha was 0.879, which shows good reliability.

#### Programming Teaching Scale

Similarly, the Programming Teaching Scale was adapted from the OECD (2009). On the basis of the original questionnaire, we have added relevant features of programming education. The scale consists of 22 items, including programming teaching methods and programming teaching management. Participants evaluated the statements using a 5-point Likert scale, where 1 means *completely disagree*, and 5 means *strongly agree*. The score ranges between 21 and 105: the higher the score, the better the programming teaching level. In the initial test of 160 students, the reliability of the scale was 0.937, and the factor loading was between 0.57 and 0.85. After formal testing, the Cronbach’s alpha was 0.939.

#### Family Socioeconomic Status Questionnaire

Based on the OECD (2009) survey of students’ family backgrounds and the conditions of information technology, we adapted the questionnaire to form the Family Socioeconomic Status Questionnaire. In particular, family cultural capital refers to the educational level of the parents, which we assigned from elementary to graduate school. Family social capital refers to the occupation of the parents. Parents’ occupations were assigned according to the occupational calculation method of the family socioeconomic status, that is, from top to bottom, according to the government or organ cadres or civil servants, business managers, ordinary employees, etc. Family economic capital was determined by the number of properties owned by the family. One point was awarded for owning an item and no points were awarded for not owing an item. The scores were then normalized for family social capital, family cultural capital and family economic capital.

### Data Analysis

In this study, all statistical analysis was performed using IBM SPSS Statistics Version 22. First, all data were electronically entered using Office 2019. After that, correlation analysis, independent sample *t*-tests, and linear regression analysis were carried out. The statistical significance level for all tests was set at *p* < 0.05. Lastly, the results were organized into tables.

### Research Process

To confirm or refute the hypotheses, data analysis was conducted in five main steps. First, we verified the correlation between creativity and the hypothetical factors to determine whether there was a relationship between variables. Next, we used independent sample *t*-tests to determine if there were gender differences in creativity and the influencing factors. Then, we divided the scores for creativity into high- and low-scoring groups and used independent sample *t*-tests to determine the difference in factor scores between these two groups, thus proving whether the average difference in different creativity scores can be inferred to the overall. After that, similarly, we divided the six hypothetical factors into high- and low-scoring groups to test whether there was a difference in creativity between these two groups for each influencing factor. Lastly, single factor regression analysis and stepwise regression analysis were carried out, and the effective prediction index and regression equation of creativity were obtained.

## Results

### Descriptive Analysis

The statistical information of participants is shown in Table 1. In terms of gender, 405 participants were male and 492 were female. In terms of age, the age of the participants was concentrated in 16–18 years old, 151 people were 18 years old, 240 people were 17 years old, and 480 people were 16 years old. Table 2 shows the mean scores and standard deviation of participants’ creativity scores and the hypothetical influencing factors. It was found that the mean total score for creativity was 3.423 (*SD* = 0.938), which indicates that the creativity of the participants inclined toward innovation. In addition, the mean score and standard deviation of other factors were: family economic capital, family social capital, family cultural capital, programming learning approach (*M* = 3.001, *SD* = 1.098), programming learning attitude (*M* = 2.959, *SD* = 1.058), programming learning engagement (*M* = 3.067, *SD* = 0.996), programming teaching method (*M* = 3.026, *SD* = 1.359) and programming teaching management (*M* = 3.569, *SD* = 1.017).

**TABLE 1:** Statistical analysis table of basic information.

	Number	Percentage (%)
Male	405	47.59%
Female	492	52.41%
16	480	56.41%
17	220	25.85%
18	151	17.74%

**TABLE 2:** Means and standard deviations for each factor.

Factor	Mean	*SD*
Creativity	3.423	0.938
Programming learning approach	3.001	1.098
Programming learning attitude	2.959	1.058
Programming learning engagement	3.067	0.996
Programming teaching method	3.026	1.359
Programming teaching management	3.569	1.017

### Relationship Between Creativity and Influencing Factors

Table 3 presents the relationships between creativity and its influencing factors in this study. The results of Pearson-correlation analysis show that there is a strong relationship between learner’s creativity and these factors: learners’ gender (*r* = –0.091, *P* = 0.008), family economic capital (*r* = 0.144, *P* < 0.001), programming learning approach (*r* = 0.330, *P* < 0.001), programming learning attitude (*r* = 0.687, *P* < 0.001) and programming learning engagement (*r* = 0.447, *P* < 0.001), programming teaching management (*r* = 0.172, *P* < 0.001) and programming teaching method (*r* = –0.084, *p* = 0.014) Among them, family economic capital, programming learning approach, programming learning attitude, programming learning engagement, programming teaching management and creativity are positively correlated, programming teaching method and creativity are negatively correlated. Family cultural capital (*r* = –0.028, *p* = 0.419) and family social capital (*r* = 0.057, *p* = 0.096) were not found to be correlated with creativity.

**TABLE 3:** Results of correlation analysis of creativity and influencing factors.

Hypothetical influencing factors	Creativity	
	Correlation coefficient	*p*
Gender	–0.091	0.008**
Family cultural capital	–0.028	0.419
Family social capital	0.057	0.096
Family economic capital	0.144	<0.001***
Programming learning approach	0.330	<0.001***
Programming learning attitude	0.687	<0.001***
Programming learning engagement	0.447	<0.001***
Programming teaching method	–0.084	0.014*
Programming teaching management	0.172	<0.001***

* = p < 0.05; ** = p < 0.01; *** = p < 0.001.

### Gender-Related Differences in Scores for Creativity and Influencing Factors

Through correlation analysis of creativity and influencing factors, we found that gender was significantly correlated with creativity among Chinese teenagers learning computer programming. We divided scores into two groups according to gender, 405 for males and 446 for females. Then we used independent sample *t*-tests to test the differences in creativity and influencing factors between the two groups. The critical *p*-value for all tests in this study was 0.05. Table 4 shows a significant difference in scores for creativity between male and female learners (*t* = 2.616, *p* = 0.009). We also used independent sample *t*-tests to determine whether there was a significant difference between females and males regarding factors influencing creativity. The results show an extremely significant difference in programming learning approach (*t* = 4.268, *p* < 0.001) and programming learning attitude (*t* = 5.618, *p* < 0.001). Gender difference was not found for other factors. In terms of family cultural capital (*t* = 0.799, *p* = 0.425), family social capital (*t* = 0.249, *p* = 0.804), family economic capital (*t* = 0.500, *p* = 0.617), programming learning engagement (*t* = –1.597, *p* = 0.111), programming teaching method (*t* = –1.627, *p* = 0.104) and programming teaching management (*t* = –1.580, *p* = 0.114), no gender difference was found.

**TABLE 4:** Gender-related differences in scores for creativity and influencing factors.

Factor	Mean		Mean gap	*t*	*P*
	Male (*N* = 405)	Female (*N* = 446)			
Creativity	3.506	3.337	0.170	2.616	0.009
Family cultural capital	0.029	–0.026	0.055	0.799	0.425
Family social capital	0.009	–0.009	0.017	0.249	0.804
Family economic capital	0.018	–0.016	0.348	0.500	0.617
Programming learning approach	3.175	2.855	0.320	4.268	<0.001
Programming learning attitude	3.172	2.766	0.405	5.618	<0.001
Programming learning engagement	3.009	3.112	–0.110	–1.597	0.111
Programming teaching method	2.946	3.099	0.152	–1.627	0.104
Programming teaching management	3.511	3.622	–0.111	–1.580	0.114

### Differences in Factor Scores Between Groups With High and Low Scores for Creativity

Table 5 shows the differences in factor scores between groups with high and low scores for creativity. First, we divided the total research participants into high- and low-scoring groups for creativity; that is, the 27% who had the highest scores for creativity became the high-scoring group, and the 27% with the lowest scores became the low-scoring group. Each group comprised 230 data sets. Second, we used independent sample *t*-tests to determine whether there were differences among the influencing factors. The results showed that the *p*-values of family economic capital (*p* < 0.001), programming learning approach (*p* < 0.001), programming learning attitude (*p* < 0.001), programming learning engagement (*p* < 0.001), programming teaching method (*p* = 0.001) and programming teaching management (*p* < 0.001) were all less than or equal to 0.01, and there were significant differences between the two groups. Accordingly, we can conclude that there is a causal relationship between these factors and creativity in this context. Furthermore, the difference between the high- and low-scoring groups was extremely significant for programming learning approach, programming learning attitude, and programming learning engagement: the mean gap was 1.043, 1.741, and 0.900, respectively. Since there were no significant differences in family cultural capital (*p* = 0.657) and family social capital (*p* = 0.076) between high- and low-scoring groups (*p* = 0.05), they were not considered as influencing factors for creativity.

**TABLE 5:** Differences in factor scores between groups with high and low scores for creativity.

Hypothetical influencing factor	Hypothetical factor (*M*)		Mean gap	*t*	*P*
	High-scoring group	Low-scoring group			
Family cultural capital	0.044	0.003	0.411	0.445	0.657
Family social capital	0.028	–0.140	0.167	1.777	0.076
Family economic capital	0.203	–0.170	0.373	3.709	<0.001
Programming learning approach	3.522	2.479	1.043	9.833	<0.001
Programming learning attitude	3.843	2.102	1.741	19.536	<0.001
Programming learning engagement	3.517	2.616	0.900	8.757	<0.001
Programming teaching method	2.810	3.250	–0.450	–3.270	0.001
Programming teaching management	3.750	3.350	0.400	3.797	<0.001

### Differences in Creativity Between Groups With Low and High Scores for Hypothetical Factors

We also verified whether the differences in the scores for hypothetical factors led to differences in the creativity of Chinese teenagers of computer programming. To achieve this, the scores for each hypothetical influencing factor were divided into three groups. The highest-scoring 27% of the total sample was the high-scoring group, and the lowest-scoring 27% was the low- scoring group, with 230 data sets each. The results, presented in Table 6, indicate differences in the creativity of high- and low-scoring groups for family economic capital, programming learning approach, programming learning attitude, programming learning engagement, programming teaching management and family cultural capital. Among them, there is relatively small difference of creativity in family cultural capital, and no difference in family cultural capital score (*p* = 0.042) and programming teaching method (*p* = 0.075). Thus, family economic capital, programming learning approach, programming learning attitude, programming learning engagement, programming teaching method and family cultural capital can be regarded as important conditions for developing the creativity of Chinese teenagers involved in programming education.

**TABLE 6:** Differences in scores for creativity between groups with high and low scores for hypothetical factors.

Hypothetical influencing factor	Creativity (*M*)		Mean gap	*t*	*P*
	High-Scoring group	Low-Scoring group			
Family cultural capital	3.288	3.468	–0.180	–2.035	0.042
Family social capital	3.491	3.380	0.116	1.346	0.179
Family economic capital	3.632	3.290	0.342	3.766	<0.001
Programming learning approach	3.864	3.116	0.748	7.977	<0.001
Programming learning attitude	4.242	2.707	1.534	18.941	<0.001
Programming learning engagement	3.886	3.045	0.841	8.633	<0.001
Programming teaching method	3.511	3.677	–0.166	–1.783	0.075
Programming teaching management	3.752	3.271	0.480	5.111	<0.001

### Analysis of the Influence of Influencing Factors on the Creativity of Chinese Teenagers Participating in Programming Education

The results presented above suggested that student gender, family economic capital, family cultural capital, programming learning approach, programming learning attitude, programming learning engagement, and programming teaching method and programming teaching management are the important factors influencing the creativity of Chinese teenagers participating in programming education. Using these eight factors as independent variables and creativity as a dependent variable, we conducted a single factor regression analysis, the results of which are presented in Table 7.

**TABLE 7:** Results of univariate linear regression—eight hypothetical influencing factors and creativity.

Hypothetical influencing factors	Constant (B)	Coefficient (Beta)	*R* ^2^	*F*	*P*
Family cultural capital	–0.026	–0.028	0.001	0.654	0.419
Family economic capital	0.134	0.144	0.021	17.875	<0.001
Programming learning approach	0.281	0.330	0.109	103.878	<0.001
Programming learning attitude	0.608	0.687	0.472	758.799	<0.001
Programming learning engagement	0.420	0.447	0.199	211.472	<0.001
Programming teaching method	–0.058	–0.088	0.008	6.239	0.013
Programming teaching management	0.158	0.172	0.030	25.820	<0.001
Gender	–0.170	–0.091	0.008	7.052	0.008

Of the eight factors, programming learning attitude had the greatest impact on creativity (*R*^2^ = 47.2%). Programming learning engagement (*R*^2^ = 19.9%), programming learning approach (*R*^2^ = 10.9%), programming teaching management (*R*^2^ = 3.0%), family economic capital (*R*^2^ = 2.1%), gender (*R*^2^ = 0.8%), programming teaching method (*R*^2^ = 0.8%) and family cultural capital (*R*^2^ = 0.1%) had less impacts on creativity. Thus, many factors influence the creativity of Chinese teenagers engaged in programming education, but these factors differ in their significance.

Similar to Liu et al.’s (2021) research methodology, the analysis also produced the following results: first, the regression equation for family economic capital, programming learning approach, programming learning attitude, programming learning engagement, programming teaching method, programming learning management and creativity is significant. Second, although family cultural capital is an influencing factor for creativity, the regression fit is not good (*R*^2^ = 0.001), and the equation is not significant (*p* = 0.419 > 0.05). Consequently, the effect size of family cultural capital is low. Finally, the significance of gender falls somewhere between the above two situations (*R*^2^ = 0.008, *p* = 0.008).

Multiple stepwise linear regression analysis was performed using the same eight factors as independent variables and creativity as the dependent variable, and the results are schematized in Table 8. Only four independent variables were retained: programming learning attitude, programming learning engagement, programming learning approach, and family economic capital. Except family economic capital (*p* = 0.003), the *p*-values of other three independent variables were less than 0.01. The variance inflation factors for these four variables were 1.868, 1.352, 1.551, and 1.031, respectively, suggesting that multicollinearity was not present. It can be concluded that in the context of programming education for Chinese teenagers, programming learning attitude, programming learning engagement, programming learning approach and family economic capital are the important influencing factors and effective predictors of learners’ creativity.

**TABLE 8:** Results of stepwise regression analysis: eight hypothetical influencing factors and creativity.

Hypothetical influencing factors	Standard coefficient	Non-standard coefficient	Standard error	*t*	*P*
Constant		0.966	0.099	9.786	<0.001
Programming learning attitude	0.521	0.461	0.026	17.622	<0.001
Programming learning engagement	0.246	0.231	0.026	8.889	<0.001
Programming learning approach	0.148	0.126	0.023	5.347	<0.001
Family economic capital	0.068	0.064	0.022	3.014	0.003

The regression equation is


Y=⁢0.461×X⁢1+0.231×X⁢2+0.126×X⁢3⁢+0.0684×X⁢4+0.966

Where, Y represents the creativity of a Chinese teenagers who is learning computer programming, X_1_ represents their attitude toward learning how to program, X_2_ represents their learning engagement, X_3_ represents their learning approach, and X_4_ represents their family’s economic capital. In addition, the *R*^2^ of the regression model is 0.488; that is, the total explanatory rate of the three independent variables to the dependent variable is 48.8%. The equation is significant.

## Discussion

Cultivating innovative talents is an important goal of education development, and improving students’ creativity has become the focus of improving education quality. As a complex and innovative curriculum, programming education is expected to support students’ creative development. More and more studies have confirmed that programming education is related to students’ creativity. However, whether programming education can promote learners’ creativity may be influenced by other factors. Therefore, it is urgent and meaningful to explore what factors affect the creativity development of students in the context of programming education in China. Based on the existing literature, this study explores how the programming learning characteristics of learners, teachers’ teaching, family socioeconomic status and student’s gender affect students’ programming creativity, thus to cope with this demand. This research is carried out in the real background of Chinese programming education, so that we have a preliminary overall understanding of the factors influencing creativity in the background of programming learning.

## Conclusion

The results of this study provide partial supports for the hypothesis constructed. The research draws the following conclusions: First, in the context of this research, the individual factors of learners, including learners’ attitudes, engagement and approach in programming learning, as well as gender differences, have an important impact on students’ creativity. Second, family economic and cultural capital can influence students’ creativity, while social capital has no influence on students’ creativity development. Third, teachers’ programming teaching including teaching methods and teaching management has little influence on students’ creativity. Moreover, the four independent variables of students’ programming learning attitude, learning engagement, learning approach and family economic capital are more important factors influencing students’ creativity, from which to build the regression equation to help predict the development of Chinese youth programming learners’ creativity tendency. The higher a programming learner scores in these four aspects, the higher level of his or her creativity. As a result, this study comprehensively considers the three main aspects that may affect students’ creativity, including learners, teachers and families. The influence of these variables on students’ programming creativity was explained, so as to provide inspiration for programming education and students’ creative development.

### Individual Learning Characteristics and the Influence of Gender on Creativity in Programming Education

Firstly, in the context of Chinese programming education, the direct factors that affect the creativity development of Chinese teenagers are some individual characteristics in learning, including the programming learning attitudes, learning engagement and learning approach. This result is consistent with other studies. For example, in the aspect of learning attitude, Kirton (1976) found that individual’s cognitive attitude is one of the main factors determining different creative tendencies. Amabile et al. (2005) also believed that positive emotional attitudes were positively correlated with creativity. As for the influence of programming learning engagement on creativity, Denner et al. (2012) found that when middle school students create diversified programming games independently, the innovation of the programming games they completed is different due to the influence of personal involvement and knowledge reserve. In terms of programming learning approach, the problem-based, project-based and game-based learning approaches are more suitable for programming learning and conducive to the development of creativity (Tomos et al., 2017; Chis et al., 2018; Gunay et al., 2019), while memorizing and traditional learning approaches are not suitable for programming learning (Wu et al., 2012; Zheng and Huang, 2019).

Secondly, in the context of Chinese programming education, learner gender affects both creativity and learning how to program. This result is consistent with existing research. There are significant differences between male learners and female learners in learning (Peng, 2019), male learners have more active learning opportunities and experiences than female learners (Brophy and Good, 1970). A study of gender differences in creative thinking also found that the areas of the brain associated with semantic cognition, rule learning and decision making were more active in men than in women, and divergent thinking easily activated (Abraham et al., 2014), this shows that boys have certain advantages in creative activities. At the same time, this conclusion is also caused by the characteristics of programming learning. The program itself is highly abstract and strict logical, and easy to cause female learners’ fear and lack of interest, male learners are significantly more interested in programming learning than female learners (Sun and Li, 2019). Many scholars have proposed that programming teaching design and practice should take into account gender differences, so that all students can effectively participate in programming learning (Becker et al., 2019; Wee and Yap, 2021).

### The Influence of Family Factors on Creativity in Programming Education

Firstly, in the context of programming education in China, family economic capital of teenagers is an important influence on the development of creativity. Combined with the above introduction, although the programming teaching in public schools in China is facing many development difficulties at present, the programming education companies are developing rapidly, which carry out interest oriented and specialty oriented programming education in a profit-making way. They established programming curriculum system from early childhood to senior high school students. This condition led to the investment of a large number of family economic capital. Some studies have also confirmed that families with high economic capital are more likely to provide good learning environment and educational resources for their children (Carvalho, 2016). In contrast, children from families with lower economic status are more likely to face more stress and hardship (Conger et al., 2010). Hence, teenagers with high family economic capital are more likely to study programming education courses and to dedicate more time to them (Zhou and Wang, 2014).

Secondly, the family cultural capital of Chinese teenagers of programming have influence on creativity. This result echoes that of Zhu (2013), who suggested that cultural environment affects innovative behaviors. Studies notes that academically successful parents are more likely to expose their children to rich resources and challenging classes (Woo et al., 2021), and participate in intellectual activities with their children, thereby indirectly supporting children’ creative development (Jankowska and Maciej, 2018). Such support is not only positive affirmation in attitude, but also economic, cultural and other aspects of support. For example, study by Liu and Morgan (2016) have shown that parents with higher cultural capital are more effective in guiding their children. They can provide children with more material, cultural knowledge, skills and other aspects of support, students’ learning motivation, and achievement are relatively high (Chiu and Chow, 2010). Therefore, parents with higher family cultural capital are more likely to accept programming, understand and be familiar with programming, and thus provide educational guidance and support in many aspects for their children’s programming learning and creativity development (Kong, 2017; Kong and Wang, 2021).

Finally, differently from the hypothesis, the family social capital of these Chinese young adults had no obvious influence on their creativity. The likely explanation of this finding is that family social capital mostly plays a greater role in supporting children who are about to be employed (Peng, 2019). In contrast, the participants in this study were upper secondary school students in China. The place where they learn programming and develop creativity is in the classroom, they get more support from teachers and parents in terms of knowledge. This is consistent with Lareau’s view, he pointed out that student’s learning is extremely complicated and advantages of social class do not necessarily lead to good educational results (Zhou and Wang, 2015). As a result, when students learn complex programming knowledge, their family’s social capital is often not directly related to creative development.

### The Impact of Teacher Teaching on Creativity in Programming Education

Unlike other studies, this study found that the current teacher’s teaching had less impact on participants’ creativity. This needs to be explained in combination with the current development of programming education in China. China has issued a new generation of artificial intelligence development plan, which clearly proposes to vigorously popularize programming education, programming education has received unprecedented policy support. However, in the actual teaching of public schools in China, programming courses are not as important as major subjects like English and math. It was not included in the heavyweight exam. In addition, although numerous studies have revealed that programming teaching should build student-centered classrooms (Wang et al., 2017; Ramirez et al., 2018). Nevertheless, teachers are better at knowledge transfer teaching at present, and their programming teaching methods, teaching experience and teaching innovation also need to be improved (Huang and Huang, 2017; Ohashi et al., 2018). All these reasons have exposed the plight of programming education in promoting the development of creativity in China at present, which is in urgent need of breakthrough.

### Implications

From the perspective of promoting the development of programming education, this study, respectively, discusses the relationship between creativity and the family factors, the programming teaching factors, the students’ learning factors and gender of programming learners, which provides necessary theoretical support and practical basis for programming education and learners’ creativity. First of all, the most crucial is that teachers need to design creative programming learning activities scientifically according to learners’ learning characteristics and rules to cultivate learners’ creativity. So as to promote learners to carry out understanding-based, inquiry-based, and project-based programming learning. Secondly, promoting learners’ creativity by programming education, attention should also be paid to gender differences. Programming teaching design and practice should take into account gender differences, so that all students can effectively participate in programming learning. Thus, the gender difference in programming learners’ creativity provides an important basis for teachers to design programming learning content and organize teaching activities reasonably. Finally, it will be the future trend to cultivate learners’ creativity through programming education from out-of-school specialty education supported by family economic and cultural capital to the popularize education in school. Therefore, it is necessary to explore the new programming education ecological environment of home-school co-education and on-and-off campus joint education, so as to provide fair, continuous and seamless opportunities and conditions for every student to develop their creativity through programming learning.

## Limitations and Directions for Future Research

While the present study has yielded findings that have implications, we recognize that its design is not without limitations. The first limitation is that, for reasons of time, money, and the convenience of the researchers, the data for this study were all from students in a single specific city. Although the city is one of the fastest developing cities in programming education in China, it may not be easy to extend the results to other regions and students from different backgrounds. The second limitation is that the generalizability evaluation of influencing creativity in this study relied on the nine factors analyzed above and not on considering the impact of programming education policies on the creativity of learners. Therefore, in future research on programming education in China, we will consider other influencing factors and expand the sampling region. In conclusion, more data and studies on other influencing factors in the future will further validate and complement the findings presented here.

## Data Availability Statement

The original contributions presented in the study are included in the article/supplementary material, further inquiries can be directed to the corresponding author/s.

## Author Contributions

JL and YZ designed the research. XS and MS performed the literature search and data analysis. JL, YZ, XS, and MS wrote, reviewed, and edited the manuscript. XL, JC, ZL, and FX reviewed and edited the manuscript. All authors contributed to the article and approved the submitted version.

## Conflict of Interest

The authors declare that the research was conducted in the absence of any commercial or financial relationships that could be construed as a potential conflict of interest.

## Publisher’s Note

All claims expressed in this article are solely those of the authors and do not necessarily represent those of their affiliated organizations, or those of the publisher, the editors and the reviewers. Any product that may be evaluated in this article, or claim that may be made by its manufacturer, is not guaranteed or endorsed by the publisher.
